# Sonic hedgehog signaling regulates mode of cell division of early cerebral cortex progenitors and increases astrogliogenesis

**DOI:** 10.3389/fncel.2014.00077

**Published:** 2014-03-11

**Authors:** Geissy L. L. Araújo, Jessica A. M. Araújo, Timm Schroeder, Adriano B. L. Tort, Marcos R. Costa

**Affiliations:** ^1^Brain Institute, Federal University of Rio Grande do NorteNatal, Brazil; ^2^Department of Biosystems Science and Engineering, Cell Systems Dynamics, ETH ZurichBasel, Switzerland

**Keywords:** cerebral cortex development, progenitor cells, sonic hedgehog (SHH), mode of cell division, neurogenesis, gliogenesis, cell survival, astrocytes

## Abstract

The morphogen Sonic Hedgehog (SHH) plays a critical role in the development of different tissues. In the central nervous system, SHH is well known to contribute to the patterning of the spinal cord and separation of the brain hemispheres. In addition, it has recently been shown that SHH signaling also contributes to the patterning of the telencephalon and establishment of adult neurogenic niches. In this work, we investigated whether SHH signaling influences the behavior of neural progenitors isolated from the dorsal telencephalon, which generate excitatory neurons and macroglial cells *in vitro*. We observed that SHH increases proliferation of cortical progenitors and generation of astrocytes, whereas blocking SHH signaling with cyclopamine has opposite effects. In both cases, generation of neurons did not seem to be affected. However, cell survival was broadly affected by blockade of SHH signaling. SHH effects were related to three different cell phenomena: mode of cell division, cell cycle length and cell growth. Together, our data *in vitro* demonstrate that SHH signaling controls cell behaviors that are important for proliferation of cerebral cortex progenitors, as well as differentiation and survival of neurons and astroglial cells.

## Introduction

Members of the Hedgehog (HH) family of proteins have been involved in a plethora of processes in the developing embryo (Ingham and McMahon, 2001). In vertebrates, the HH homolog Sonic Hedgehog (SHH) plays critical roles in the patterning of the developing neural tube system, limbs, axial skeleton, and other derivatives of the somites (Chiang et al., 1996). In the neural tube, SHH signaling is pivotal for the patterning of ventral structures (Echelard et al., 1993), proliferation and survival of ventral progenitors (Rowitch et al., 1999) and specification of ventral neurons, such as motoneurons in the spinal cord (Ericson et al., 1995; Roelink et al., 1995; Briscoe et al., 2000) and GABAergic interneurons in the cerebral cortex (Xu et al., 2005, 2010).

SHH is also expressed in the dorsal telencephalon during mid and late corticogenesis where it has been associated with growth of cortical structures (Dahmane et al., 2001), generation and maintenance of post-natal and adult neural stem cell pools (Machold et al., 2003; Ahn and Joyner, 2005; Palma et al., 2005; Han et al., 2008). Selective genetic deletion of SHH or its receptor Smoothened (SMO) in the dorsal telencephalon leads to decreased proliferation of progenitor cells, reduced neurogenesis and increased cell death (Komada et al., 2008), indicating a central role for SHH signaling in the control of cortical neurogenesis. However, the cellular mechanisms underlying these effects are still poorly understood.

In this study, we show that SHH affects cell survival, cell cycle progression, cell growth and mode of cell division of cortical progenitors isolated from the dorsal telencephalon at early corticogenesis. Blockade of SHH signaling using cyclopamine in cultures of embryonic day (E)13 cortices led to a premature depletion of progenitor cells and diminished astrogliogenesis, with no direct effect on neurogenesis. Conversely, stimulation of SHH pathway increased proliferation and generation of astroglial cells, but did not affect the generation of neurons. Intriguingly, we show that SHH effects on cell cycle length and mode of cell division of cortical progenitors are linked suggesting that control of cell cycle by SHH may be an important mechanism to govern the balance between proliferation and differentiation of neural cells.

## Materials and methods

### Animals

C57/Bl/6J and Tau-GFP (Tucker et al., 2001) mice were maintained on a 12-h (7:00 or 19:00 h) light-dark cycle. The day of the vaginal plug was considered as E0 and the day of birth as P0. All experimental procedures were done in accordance with the Society for Neuroscience and were approved by our institutional animal care and utilization committees.

### Primary cell culture

Embryonic brains were isolated from E13 timed pregnant mice. The lateral portion of the dorsal telencephalon was dissected and dissociated as previously described (Costa et al., 2009). Approximately, 5 × 10^5^ cells (containing both neural progenitor cells and post-mitotic neurons) were plated on poly-D-lysine (PDL) coated glass coverslips in DMEM GlutaMax (GIBCO) supplemented with 10% fetal calf serum (FCS) (GIBCO). After 2 h, cultures were infected with low titer (<25 particles) of the retroviruses carrying the gene encoding for the green fluorescent protein GFP and treated with cyclopamine (5 μ M), SHH (5 nM), or etanol (0.05%). Twenty-four hours later, equal volume of DMEM GlutaMax supplemented with B27 (GIBCO) and the respective treatments were added, reducing the FCS concentration to 5% while keeping the same concentration of cyclopamine, SHH or etanol. After 2, 5, or 7 days, cultures were fixed with 4% PFA at room temperature (5 min) and processed for immunocytochemistry.

### Immunocytochemistry

Cell cultures were incubated in primary antibody overnight at 4°C in 0.5% of triton X-100 and 10% of normal goat serum in PBS 0.1M. Primary antibodies used were anti-MAP2 (mouse IgG1, SIGMA 1:1000), anti-GFP (chicken, AvesLab 1:500) anti-Ki67 (rat, DAKO 1:50), anti-GFAP (rabbit, DAKO 1:500). Fluorescent secondary antibodies were used according to the manufacturer's recommendations (Life Technologies). Nuclei were visualized by incubating cells for 10 min with 0.1 μ g/mL DAPI (4′6′-diamidino-2-phenylindone, SIGMA) in PBS 0.1 M. Cells were mounted in Aqua Polymount (Polyscience) and analyzed using a Cell Observer equipped with epi-fluorecence and LSM 710 confocal laser scanning microscopes (Zeiss).

For quantification of cell numbers by immunocytochemistry, we randomly sampled 12 areas (measuring 143315.21 μm^2^ each) from 3 independent experiments and quantified the total number of DAPI nuclei, Ki67, GFAP, or MAP2 labeled cells.

### Clonal analysis *in vitro*

Clones were classified according to the expression of GFP and the neuronal MAP2 and astroglial GFAP markers. Immunoreactivity for these markers was revealed with secondary antibodies with different conjugated fluorophores, allowing the identification of three types of clones: pure neuronal (all cells stained for MAP2), pure glial (absence of MAP2-positive cells and immunoreactivity for GFAP), and mixed clones (at least one MAP2-positive cell and one O4 or GFAP-positive). We analyzed 578 clones from 4 independent experiments (Control: 329 clones; Cyc: 134 clones; SHH: 115 clones).

### Time-lapse video microscopy

Cell cycle parameters, cell area, mode of cell division and cell survival were analyzed by time-lapse video microscopy (Costa et al., 2008, 2011). Briefly, cultures were imaged every 4 min using a Cell Observer microscope (Zeiss) and a self-written VBA module remote controlling Zeiss AxioVision software (Rieger et al., 2009). Images were assembled into a movie using the software Timm's Tracking Tool—TTT (Rieger et al., 2009), allowing the identification and tracking of individual clones. Mode of cell division was classified based on the behavior of daughter cells in: Symmetric Progenitor (both daughter cells continue to proliferate), Asymmetric (one daughter cell continues to proliferate and the other becomes post-mitotic), or Symmetric Terminal (both daughter cells become post-mitotic). Cell cycle length was measured as the time spanned by proliferating cells between their generation and division. Cell size was measured as the area covered by the cell's soma (in μ m^2^) 10 min prior to division. Cell survival was quantified every 12 h for each cell lineage. Briefly, the number of cells alive at 12, 24, 36, 48, 60, 72, and 84 h was divided by the total number of cells generated before these time-points within individual clones.

### Statistical analysis

Data were derived from at least 3 independent batches of cell culture. In each experiment, cells were isolated from the dorsal telencephalon of 5–6 embryos and we analyzed at least 2–3 coverslips from each condition (control, cyclopamine, and SHH) per experiment. The total number of cells or clones analyzed is provided throughout the results section.

Statistical analyses were made using the software GraphPad Prism version 5 (GraphPad) and MATLAB. Data in the graphics are presented as Mean ± Standard Error of the Mean (SEM). For statistical significance we considered ^*^p < 0.05, ^**^p < 0.01 and ^***^p < 0.001, using *t*-test, One-Way ANOVA followed by Tuckey or Dunnet *post-hoc* tests, or Two-Way ANOVA followed by Bonferroni *post-hoc* test, as indicated in the figure legends.

## Results

### SHH signaling affects the generation of glial cells from dorsal telencephalic progenitors

**Figure 1:**
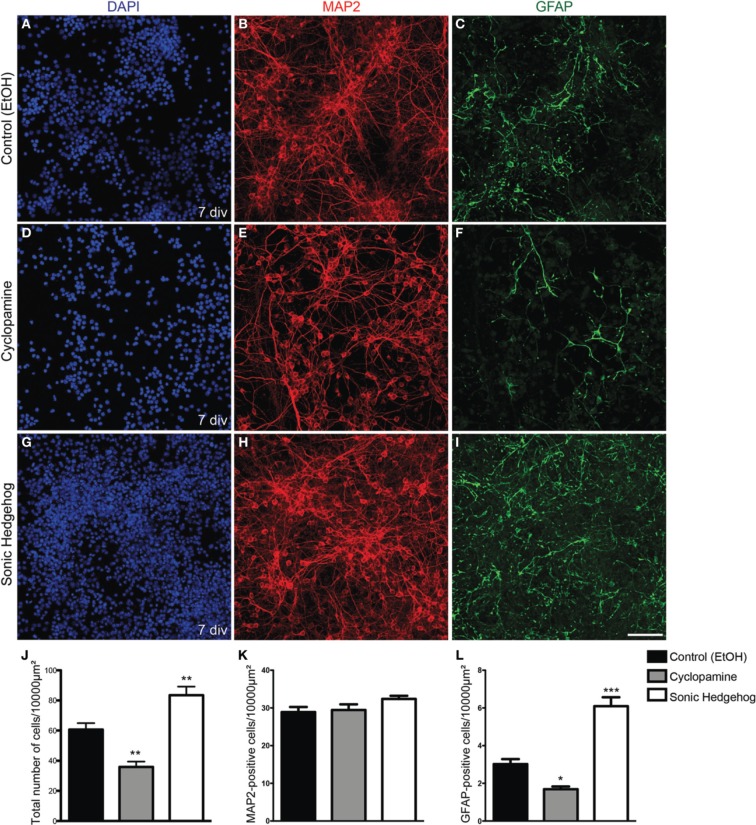
**Increased cellularity in cultures treated with SHH. (A–I)** Images of E13 cortical cell cultures treated with EtOH **(A–C)** control, cyclopamine **(D–F)**, or SHH **(G–I)**. Cultures were immunolabeled after 7 div using antibodies against GFAP (green) and MAP2 (red) and stained with DAPI (blue). Note the reduction in GFAP-expressing cells in cultures treated with cyclopamine **(F)** and the increase in this population upon SHH exposure **(I)**, compared to control **(C)**. **(J–L)** Quantification of the total number of cells **(J)** MAP2-positive neurons **(K)** or GFAP-positive astrocytes **(L)** after 7 div. ^*^p < 0.05, ^**^p < 0.01, ^***^p < 0.001, One-Way ANOVA followed by Dunnet *post-hoc* test. Calibration bar: 100 μm.

Progenitor cells in the dorsal telencephalon express SHH targets such as GLI genes at early and mid-neurogenesis (Dahmane et al., 2001; Komada et al., 2008). However, the effects of this signaling in the fate of cortical progenitors are poorly understood. To test whether SHH signaling could influence the fate of early cortical progenitors, we treated cultures of cortical progenitors with recombinant SHH or cyclopamine, a HH signaling pathway inhibitor (Chen et al., 2002). After 7 days *in vitro* (div), we observed that cultures treated with SHH displayed an increase in the number of cells (Figure 1). While the amount of cells reactive for the neuronal marker MAP2 (microtubule-associated protein 2) was not affected (Figure 1K), the total number of cells and cells reactive for the astrocyte marker GFAP (glial-fibrilliary acidic protein) was higher in SHH treated cultures as compared to controls. In contrast, cultures treated with cyclopamine exhibited lower numbers of GFAP-expressing cells and total number of cells than controls (Figures 1J,L).

**Figure 2:**
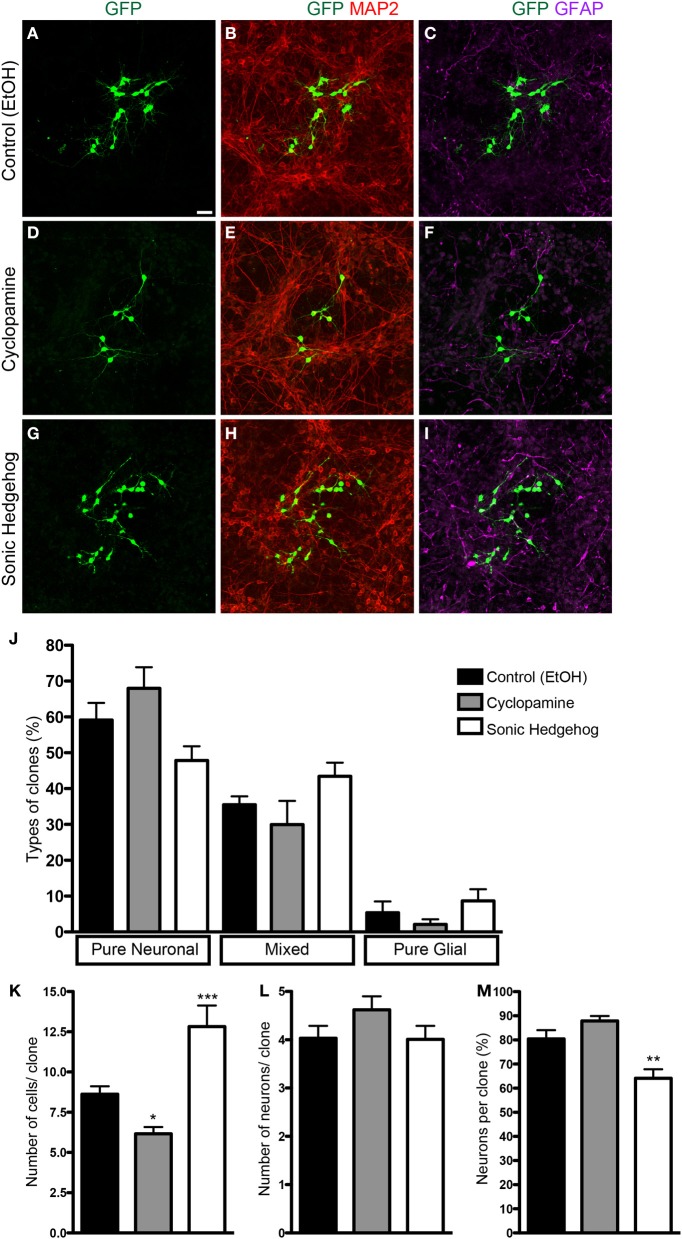
**Clonal analysis using retroviral vectors. (A–I)** Images of cortical cell cultures after 7 div, immunolabeled with antibodies against GFP (green), MAP-2 (red), and GFAP (magenta). **(J)** Quantification of the types of clones in different conditions. **(K–M)** Quantifications of total number of cells **(K)**, number of neurons **(L)**, and percentage of neurons **(M)** per clone. Note that the effects in the number of cells are not accompanied by changes in the number of neurons per clone **(K,L)**. ^*^p < 0.05, ^**^p < 0.01, ^***^p < 0.001, One-Way ANOVA followed by Dunnet *post-hoc* test (Control: 329 clones; Cyc: 134 clones; SHH: 115 clones; *n* = 4 independent experiments). Calibration bar: 50 μm.

To isolate the effects of SHH in progenitor cells from those in post-mitotic neurons isolated in our cell culture preparation, we used retroviral labeling of cortical progenitors after 2 h *in vitro* and analyzed clone size and composition after 7 div (Figure 2). Since only mitotic progenitor cells incorporate the retroviral genome carrying the reporter gene (Price et al., 1987), the use of a low number of retroviral particles allows the identification of cells derived from a single progenitor, i.e., a clone. We could observe that the frequency of pure neuronal, mixed and pure glial clones was not significantly affected by SHH or cyclopamine (Figure 2J). However, the number of cells per clone was significantly decreased in cultures treated with cyclopamine, and increased with SHH (Figure 2K). Interestingly, the mean number of neurons per clone was not affected (Figure 2L), suggesting that SHH signaling increases the number of undifferentiated and/or macroglial cells, leading to a reduction in the percentage of neurons per clone (Figure 2M).

**Figure 3:**
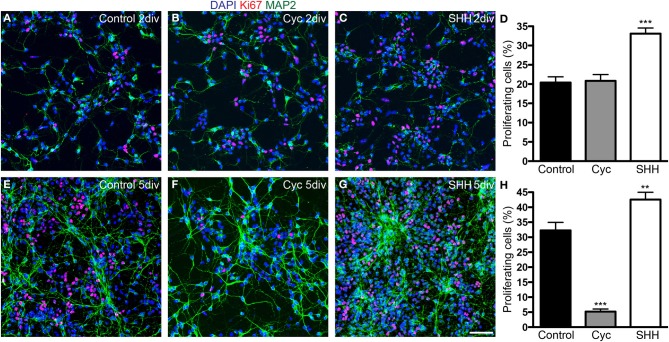
**SHH signaling increases the number of proliferating cells. (A–C,E–G)** Images of cortical cell cultures after 2 **(A–C)** or 5 div **(E–G)** immunolabeled with antibodies against Ki67 (red) and MAP-2 (green). Cell nuclei are stained with DAPI (blue). **(D,H)** Quantification of Ki67-expressing cells. ^**^p < 0.01, ^***^p < 0.001, One-Way ANOVA followed by Dunnet *post-hoc* test (Number of cells analyzed, 2 div—Control: 1863; Cyc: 2170; SHH: 2986; 5 div—Control: 5342; Cyc: 3015; SHH: 6170; 3 independent experiments). Calibration bar: 50 μm.

Next, we quantified the total number of cells and progenitors after 2 and 5 div. SHH treatment increased the number of both proliferating (Ki67-expressing) and non-proliferating cells after 2 div, and this effect persisted after 5 div (Figure 3). In contrast, cyclopamine did not affect the number of proliferating and non-proliferating cells at day 2 *in vitro* (Figure 3D), but led to a significant decrease in the number of proliferating cells at day 5 (Figure 3H), with no effect in the number of non-proliferating cells. These data indicate that cyclopamine is mainly affecting progenitor cells generated at late stages in culture (between 2nd and 5th day).

### SHH signaling influences cell division mode

**Figure 4:**
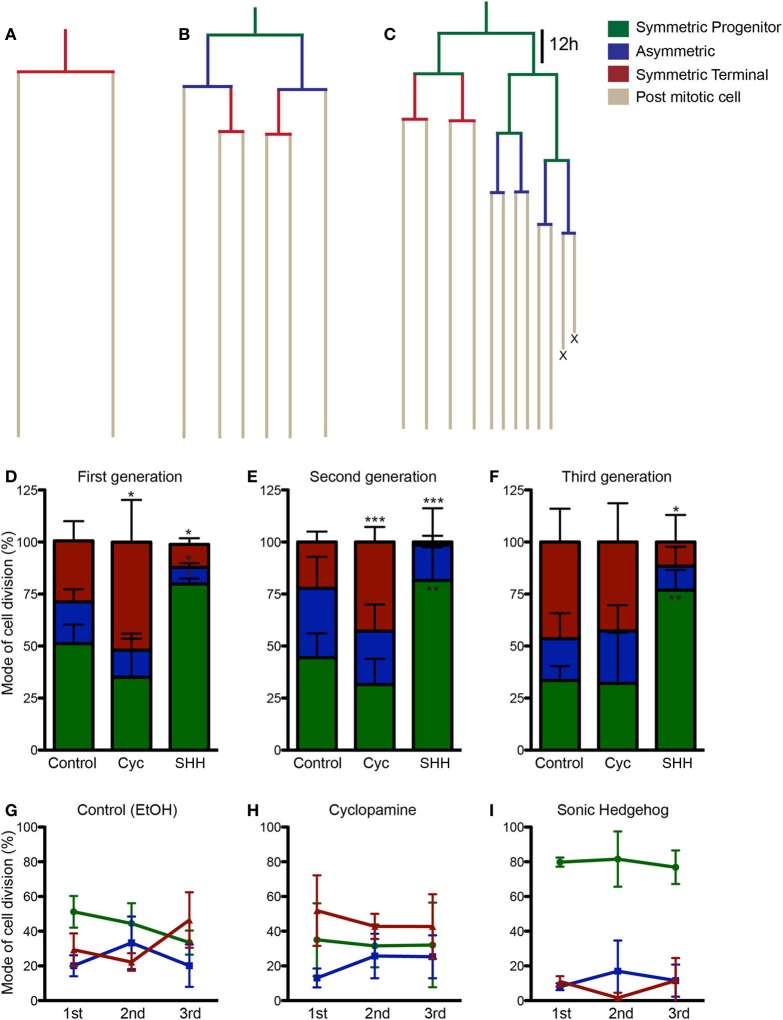
**SHH increase frequency of progenitor cell divisions. (A–C)** Examples of lineages trees from individual cortical progenitors observed in cyclopamine **(A)**, control **(B)** and SHH **(C)** treated cultures. Lines are color coded to indicate cells undergoing SP (green), As (blue), or ST (red) cell divisions. **(D–F)** Quantification of SP, As and ST cell divisions in the first **(D)**, second **(E)**, and third **(F)** cell generations. **(G–I)** Graphics showing the evolution in the frequency of SP, As and ST cell division in the lineage progression of control **(G)**, cyclopamine **(H)**, and SHH **(I)** treated cells. Note that the pattern observed between SP and ST cell divisions in control cultures is abolished in cyclopamine and SHH treated conditions. ^*^p < 0.05, ^**^p < 0.01, ^***^p < 0.001, One-Way ANOVA followed by Dunnet *post-hoc* test (Number of cells analyzed, first generation—Control: 77, Cyc: 66, SHH: 86; second generation—Control: 55, Cyc: 51, SHH: 75; third generation—Control: 60, Cyc: 27, SHH: 59).

To get a better understanding on the cellular mechanisms leading to the changes in cell population induced by SHH and cyclopamine, we next performed time-lapse video microscopy experiments. Cortical progenitor cultures were imaged every 5 min up to 7 div. Images were assembled into a movie using Timm's Tracking Tool (TTT), allowing the tracking of individual progenitor cells and its progeny (Movies 1–3). Figure 4 shows examples of common lineages trees observed in cultures treated with cyclopamine (Figure 4A), control (Figure 4B), and SHH (Figure 4C). Lineages are color coded to facilitate identification of cell division mode: symmetrically, generating two progenitor cells (Symmetric Progenitor, SP) or two post mitotic cells (Symmetric Terminal, ST); and asymmetrically, generating one progenitor and one post mitotic cell (Asymmetric, As).

We quantified the frequency of cortical progenitors undergoing each type of cell division (Figures 4D–F). In the first generation (first recorded round of cell division), we observed a significant increase in the number of SP cell divisions and a decrease in ST and As divisions after SHH treatment, whereas cyclopamine significantly increased ST cell divisions (Figure 4D). A similar trend persisted for ST and SP cell division in the second generation (progenitor cells generated from first generation cells), but with no changes in As cell divisions (Figure 4E). Interestingly, in the third generation we failed to observe any difference between control and cyclopamine treated cultures, but SHH still increased SP and decreased ST cell divisions (Figure 4F).

Next, we plotted the frequency of SP, ST, and As cell divisions per generation for each group. As previously described, we observed that cortical progenitors divided more symmetrically generating two progenitors in the first generation, but progressively shifted to generate two post mitotic cells (Figure 4G). However, both cyclopamine and SHH changed this pattern of frequency of SP and ST divisions over time (Figures 4H,I). Together, these data indicate that SHH signaling regulates cell cycle exit and reentry in cortical progenitors *in vitro*.

### Effects of SHH signaling in cell cycle length and cell growth

**Figure 5:**
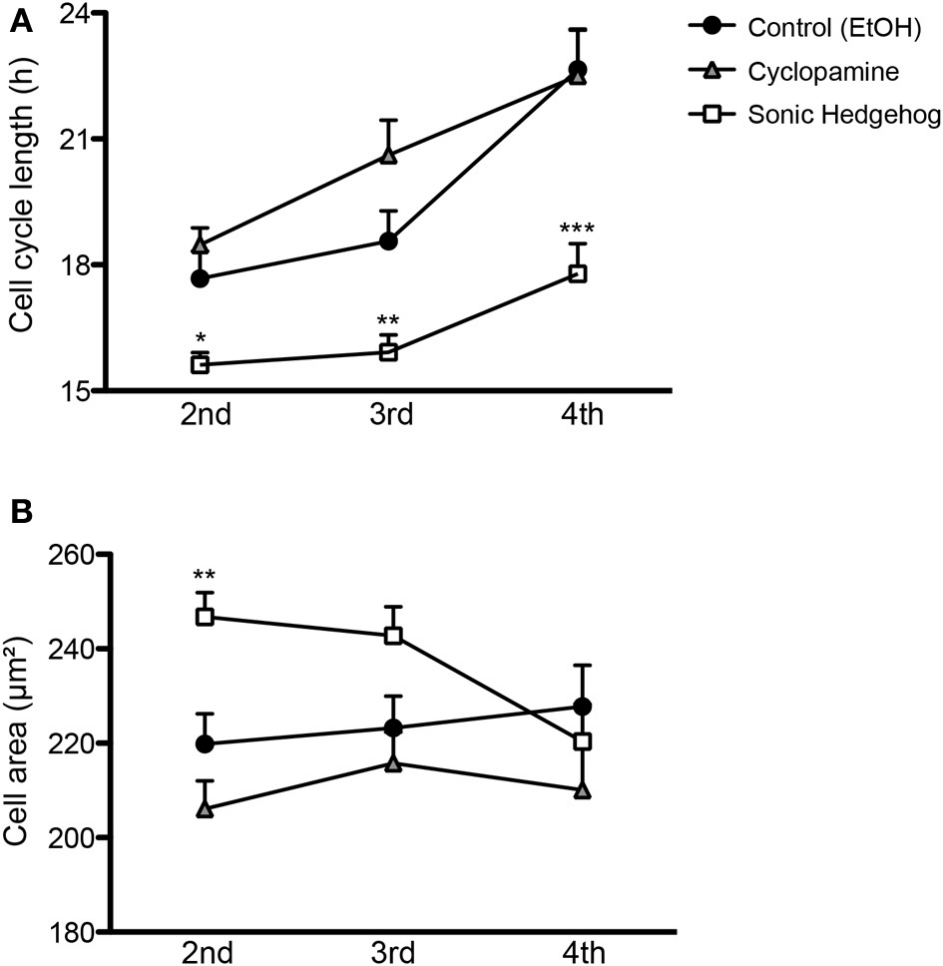
**SHH signaling influences cell cycle length and cell size of cortical progenitors. (A,B)** Quantification of cell cycle length **(A)** and cell area prior to division **(B)** of cortical progenitors in the second, third and fourth generations. ^*^p < 0.05, ^**^p < 0.01, ^***^p < 0.001, One-Way ANOVA followed by Dunnet *post-hoc* test (Number of cells analyzed, second generation—Control: 55, Cyc: 51, SHH: 75; third generation—Control: 60, Cyc: 27, SHH: 59; fourth generation—Control: 40, Cyc: 19, SHH: 157).

Cell cycle length of cortical progenitors is an important predictor for neuronal differentiation (Calegari et al., 2005; Arai et al., 2011). To test whether SHH signaling interferes with cell cycle length, we quantified the time spanned by proliferating cells in the second (between the first and second cell division), third (between the second and third cell division), and fourth (between the third and fourth cell division) generations (Figure 5). We observed that SHH treatment shortened the cell cycle in every generation, whereas cyclopamine did not affect cell cycle length (Figure 5A). These observations suggest that SHH signaling controls proliferation and differentiation of cortical progenitors by regulating the expression of cell cycle molecules.

Alternatively, cell cycle length could indirectly affect the fate of newly generated cells by interfering with cell growth, which has been related to the proliferative capacity of adult neural stem cells (Costa et al., 2011). To rule out the possibility that a shorter cell cycle could lead to a smaller cell volume prior to cell division, we measured the size of progenitor cells 10 min before cell division. We observed that SHH increased cell size only in the second progenitor generation (Figure 5B), an effect contrary to the idea that a longer cell cycle would lead to larger cells. These observations suggest that changes in cell cycle length and cell area induced by SHH are not directly correlated.

**Figure 6:**
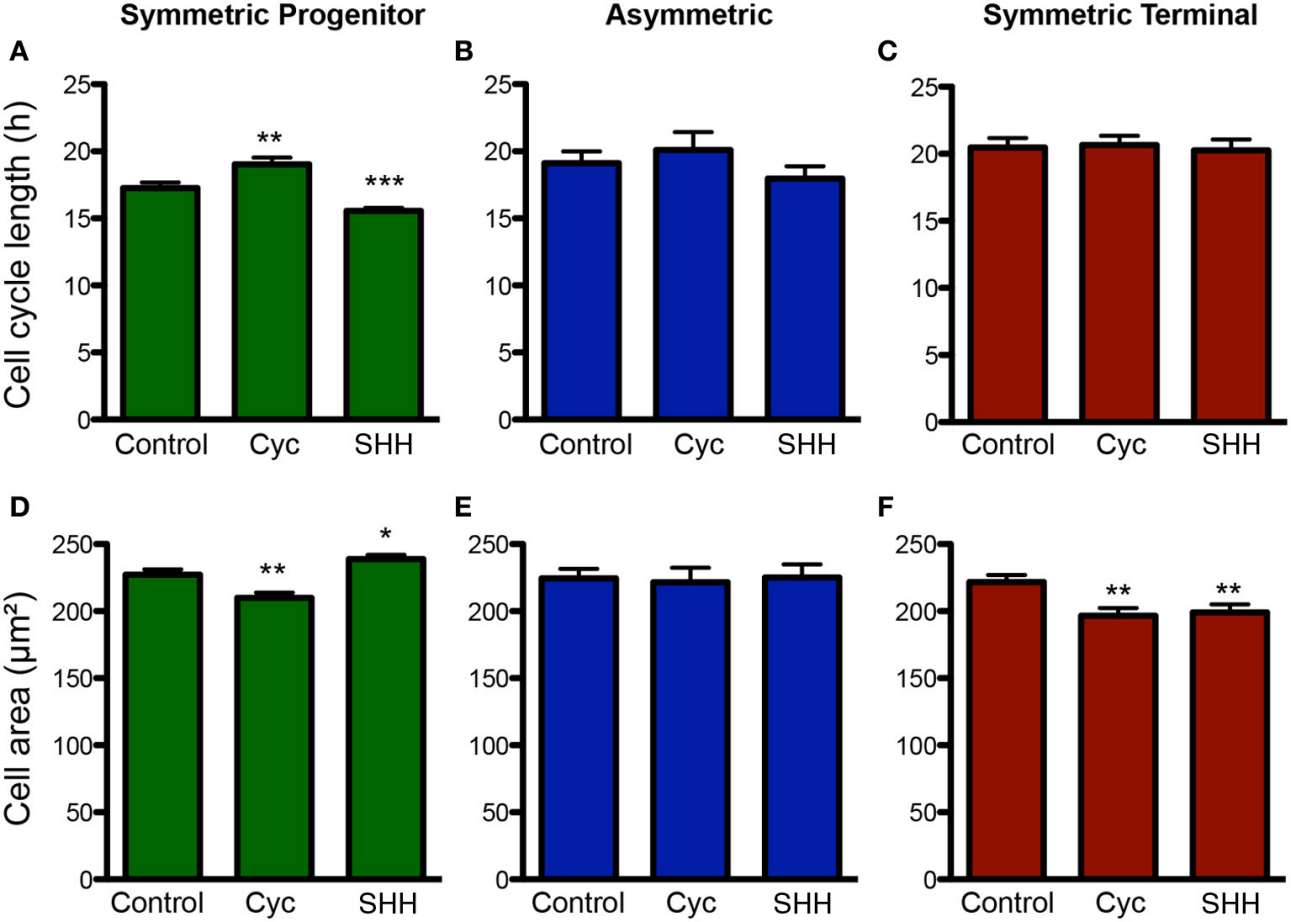
**Changes in cell cycle length and cell size are more pronounced among SP progenitors. (A–C)** Quantification of cell cycle length for progenitors undergoing SP **(A)**, As **(B)** or ST **(C)** cell divisions upon control, cyclopamine or SHH treatments. **(D–F)** Quantification of cell area for progenitors undergoing SP **(D)**, As **(E)**, or ST **(F)** cell division. ^*^p < 0.05, ^**^p < 0.01, ^***^p < 0.001, One-Way ANOVA followed by Dunnet *post-hoc* test (Number of cells analyzed, SP control: 123, SP cyc: 100, SP SHH: 224; As control: 38, As cyc: 18, As SHH: 21; ST Control: 95, ST cyc: 60, ST SHH: 45).

Next, we analyzed the effects of SHH signaling on cell cycle length and cell area of progenitors undergoing different types of cell divisions (Figure 6). Interestingly, we observed that the cell cycle length varied specifically among progenitors undergoing SP cell divisions (Figure 6A), suggesting that the capacity to continue proliferating may be directly affected by cell cycle length. Similarly, cell area of SP progenitors was also affected by treatments (Figure 6D). Contrary to the cell cycle, however, we also observed changes in the cell area of ST progenitors (Figure 6F). For both parameters, no changes were observed among As progenitors (Figures 6B,E). Taken together, these data suggest that SHH effects on the mode of cell division of cortical progenitors could be related to cell cycle length and cell growth control.

### Changes in cell cycle length and cell size are related to the mode of cell division

**Figure 7:**
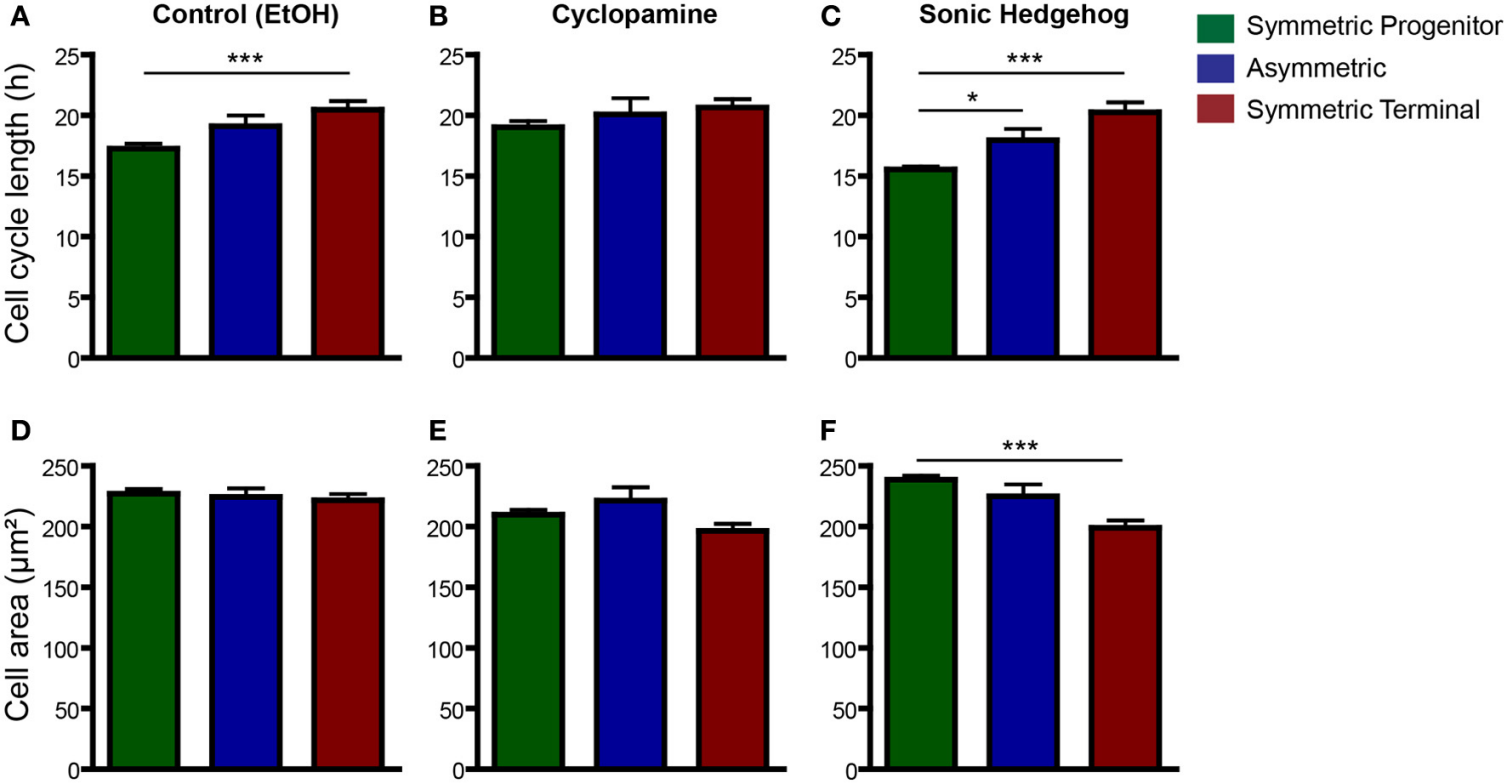
**Cell cycle length is a good predictor for SP and ST cell division. (A–C)** Same data as in Figure 6, but with plots rearranged to allow comparisons across division modes—control **(A)**, cyclopamine **(B)**, or SHH **(C)** treated cell cultures. Observe that cell cycle of ST progenitors is significantly longer than SP progenitors in control conditions **(A)**. This difference is also observed in SHH treated cultures **(C)**, but not upon cyclopamine treatment **(B)**. **(D–F)** Quantification of cell area of SP, As or ST progenitors in control **(D)**, cyclopamine **(E)**, or SHH **(F)** treated cell cultures. ^*^p < 0.05, ^***^p < 0.001, One-Way ANOVA followed by Tukey *post-hoc* test (Number of cells analyzed, SP control: 123, SP cyc: 100, SP SHH: 224; As control: 38, As cyc: 18, As SHH: 21; ST Control: 95, ST cyc: 60, ST SHH: 45).

It has recently been shown that cell fate is tightly associated with the cell-cycle machinery and that the capacity of differentiation of stem cells varies during their cell cycle (Pauklin and Vallier, 2013). To test whether cell cycle length could predict a specific mode of cell division, we next compared changes in cell cycle length among progenitors cells undergoing SP, As, or ST cell divisions (Figure 7). In controls, we found that cell cycle length of cells undergoing ST was significantly longer than that of cells undergoing SP cell divisions (Figure 7A). In cyclopamine treated cultures, this difference was abolished (Figure 7B), whereas in SHH treated cultures cell cycle length of SP, As, and ST cell divisions were significantly different (Figure 7C). In contrast, only in cultures treated with SHH we could observe a significant difference between the size of progenitors undergoing SP vs. ST cell division (Figure 7F). These data suggest that cell cycle length is a positive predictor of the mode of cell division and may be indirectly responsible for the effects of SHH signaling on the proliferation of cortical progenitors, contributing to control the number of cells in the cerebral cortex.

### Inhibition of SHH signaling increases cell death

**Figure 8:**
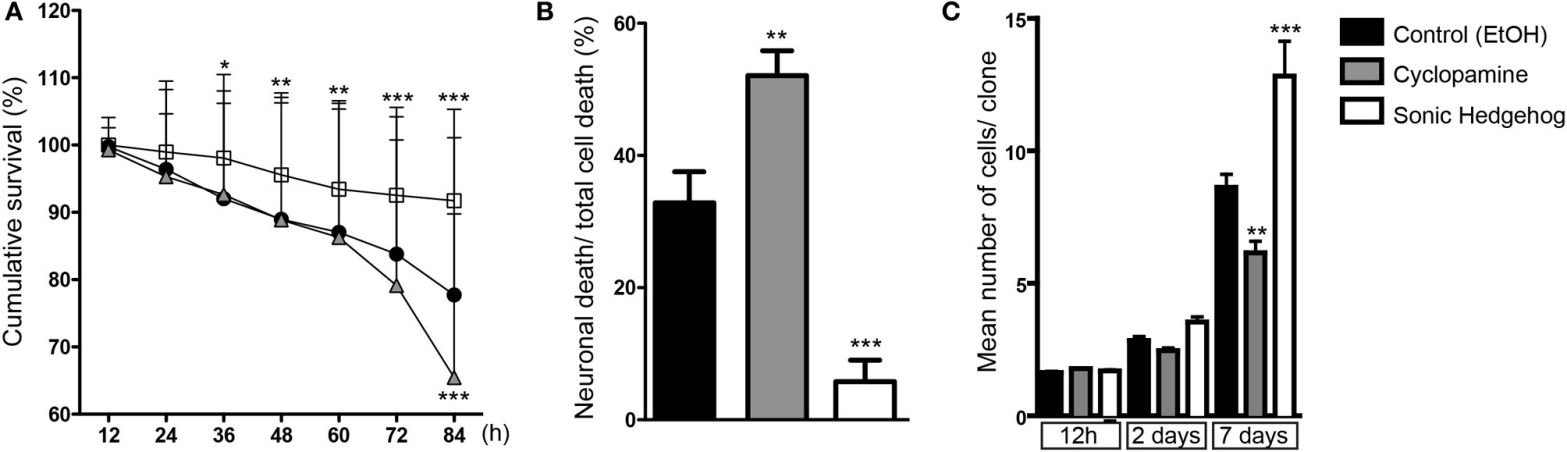
**SHH improves survival of cortical cells. (A)** Cumulative cell survival of cortical cells in cultures treated with cyclopamine (gray triangles), SHH (white squares), and control (black circles). Note that SHH increases cell survival at early time-points, whereas cyclopamine decreases cell survival at 84 h. ^*^p < 0.05, ^**^p < 0.01, ^***^p < 0.001 (row factor), Two-Way ANOVA followed by Bonferroni *post-hoc* test, number of clones analyzed, control: 137, cyc: 125, SHH: 182. **(B)** Quantification of the percentage of neuronal death among total cell death within lineages (^**^p < 0.01, ^***^p < 0.001, One-Way ANOVA followed by Dunnet *post-hoc* test). **(C)** Quantification of the mean number of cells generated from a single progenitor, e.g., clones, after 12 h, 2 and 7 days *in vitro* (*n* = 3 independent experiments; ^**^p < 0.01, ^***^p < 0.001, One-Way ANOVA followed by Tukey *post-hoc* test).

Cellular survival is another important mechanism controlling cell number during cerebral cortex development (Blaschke et al., 1996; Thomaidou et al., 1997). In order to evaluate the influence of SHH signaling on cortical cell death, we quantified the cumulative survival of cells within individual cell lineages (Figure 8). We found that SHH significantly increased cell survival after 36 h *in vitro*, and this effect persisted up to 84 h (Figure 8A). Conversely, blockade of SHH signaling by cyclopamine led to a significant decrease of cell survival at 84 h, with no detectable effects at earlier time points (Figure 8A).

We also noticed a substantial amount of neuronal (Tau-GFP) cell death in cyclopamine treated cultures compared to SHH and control (Movies 1–3). To quantify this effect, we divided the total number of GFP-expressing neurons undergoing cell death by the number of cells dying within individual cell lineages. In fact, we found that half of the cells suffering cell death in cyclopamine treated cultures were neurons (Figure 8B). Moreover, we observed a significant effect of SHH on neuronal survival, reducing about 6-fold the percentage of neurons suffering cell death.

Taken together, our data indicate that augmented SHH signaling positively affects proliferation and survival, whereas SHH blockade induces cell differentiation and reduces cell survival of both neurons and cortical progenitors. These lead to a remarkable effect on the number of cells generated by individual cortical progenitors (Figure 8C), especially after 48 h of culture when the effects of cyclopamine on proliferation (Figures 3, 4) and survival (Figure 8A) become more prominent.

## Discussion

In this *in vitro* work, we have unraveled a potential new role for SHH in the developing forebrain. Besides its well-known functions in midline formation, patterning of ventral telencephalon and specification of GABAergic interneurons (Tole et al., 2000; Xu et al., 2005, 2010; Rash and Grove, 2007), here we showed that SHH signaling controls progenitor cell proliferation, cell survival and generation of glial progenitors/astrocytes in cells isolated from the dorsal telencephalon at early corticogenesis and grown *in vitro*. Moreover, we described a strong correlation between SHH effects on cell cycle length and cellular growth and the mode of cell division, which could help understanding the molecular mechanisms regulating proliferation and differentiation in the developing nervous system.

Expression of SHH and its target genes in the dorsal telencephalon has been previously reported. Yet, studies about the functions of SHH signaling in this region have mostly concentrated at mid to late-corticogenesis (Dahmane et al., 2001; Machold et al., 2003; Palma and Ruiz i Altaba, 2004; Ahn and Joyner, 2005; Palma et al., 2005). Recently, it has been shown that conditional genetic deletion of SHH or its receptor SMO in dorsal telencephalic progenitors at early corticogenesis (around E10.5 using Emx1-Cre) leads to a reduction in the brain size, possibly caused by changes in cell proliferation and cell death in the dorsal telencephalon of conditional SHH and SMO knockouts (Komada et al., 2008). Our present results shed new light on the cellular mechanisms responsible for the effects of SHH signaling on the control of cell number in the developing cerebral cortex.

Similarly to previous work (Palma et al., 2005; Komada et al., 2008), we observed a reduction in cell proliferation after decreasing SHH activity with cyclopamine. Moreover, we showed that this effect was a consequence of a longer cell cycle, increased symmetric terminal divisions and augmented cell death. The finding that cyclopamine did not affect the number of neurons generated *in vitro*, but did affect the generation of GFAP-expressing cells indicates that SHH signaling is important to keep the progenitor pool that will become gliogenic at late corticogenesis (Costa et al., 2009). Alternatively, cyclopamine treatment could be directly interfering with specification of glial progenitors.

In an opposite direction, exposure to SHH led to a dramatic increase in proliferation and, consequently, in cell numbers. Notably, however, this increase in cell number was not caused by an enhanced generation of neurons, but was rather accompanied by an increase in the number of Ki67- and GFAP-expressing cells. This result indicates that SHH is acting to keep cells in a proliferative state and toward increasing gliogenesis. In fact, SHH signaling is involved in the generation of oligodendrocytes in the ventral telencephalon of mice (Tekki-Kessaris et al., 2001) and oligodendrocyte progenitors (OPCs) in the human embryonic brain (Ortega et al., 2013). However, our results indicate that SHH signaling may also be important for the generation of astrocytes in the cerebral cortex. Future experiments should address this possibility *in vivo*.

Our findings *in vitro* are also in accordance with previous results *in vivo* indicating that cell cycle length of cortical progenitors is an important predictor of cell fate (Calegari et al., 2005; Lange et al., 2009; Attardo et al., 2010; Arai et al., 2011). In fact, it has been shown that cortical progenitors with longer cell cycle are more likely to give rise to post-mitotic neurons after division (Arai et al., 2011). Therefore, the effects of SHH and cyclopamine treatments on cell cycle could be indirectly affecting the outcome of cell division of cortical progenitors observed here.

SHH signaling is a well-known regulator of cell cycle through transcriptional regulation of genes directly involved in cell proliferation, such as cyclin D1 and D2, proliferating cell nuclear antigen (PCNA) and N-myc (Oliver et al., 2003). In addition, it has recently been shown that SHH induces transactivation of the epidermal-growth factor receptor (EGFR) and facilitates mitogenic signaling via the ERK1/2 pathway (Reinchisi et al., 2013). Changes in cell size upon SHH or cyclopamine treatment could also affect mitogenic activity by altering protein phosphorylation levels (Meyers et al., 2006). Thus, the changes in cell cycle described here are in accordance with established functions of SHH.

An important novelty of our study, however, is the observation that cell cycle length may be a predictor for the mode of cell division. In fact, we showed that cortical progenitors dividing symmetrically to generate two progenitors have a shorter cell cycle than cells dividing symmetrically to generate two post mitotic cells under control conditions. Upon cyclopamine treatment, the cell cycle length of ST, As, or SP progenitors were virtually equal and cortical progenitors were not capable of progressing from a high SP stage (first and second generation) to a high ST stage (third generation). Conversely, treatment with SHH kept cortical progenitors in a high SP state, but due to a shorter cell cycle. Interestingly, this effect on cell cycle length was particularly evident among SP progenitors, suggesting that SHH signaling is an important mechanism to control the proliferative state of cortical progenitors.

SHH signaling is also implicated in cell survival (Komada et al., 2008; Reinchisi et al., 2013). Indeed, we showed that SHH improves while cyclopamine decreases the survival of cells. Interestingly, increased cell death in cyclopamine treated cultures was evident after 72 h, when many progenitors have already lost neurogenic potential and became gliogenic (Costa et al., 2009). Together with the absence of effect in neurogenesis showed by clonal analysis, these data *in vitro* suggest that SHH signaling regulates generation and/or survival of glia-restricted progenitors in the developing cerebral cortex.

Collectively, our results indicate that SHH signaling plays important roles in the control of cell cycle, cell growth, and mode of cell division of dorsal telencephalic progenitors at early corticogenesis, thus likely contributing to the intricate balance between proliferation and differentiation in the developing cerebral cortex. Together with SHH effects on astrogliogenesis, neuronal and progenitor cell survival, our data reveal new functions for this multipurpose morphogen.

### Conflict of interest statement

The authors declare that the research was conducted in the absence of any commercial or financial relationships that could be construed as a potential conflict of interest.

## Supplementary material

Movie 1 **Control treatment**. E13 Tau-GFP (green) cortical cells treated with ethanol and infected with retroviruses carrying the gene for the red fluorescent protein (RFP). Increased number of RFP reflects mitotic activity in the culture, whereas Tau-GFP expression reveals neuronal differentiation.

Movie 2 **Cyclopamine treatment**. E13 Tau-GFP cortical cells treated with cyclopamine and infected with retroviruses carrying the gene for RFP. Note the discrete increase in numbers of RFP and Tau-GFP cells as compared to control (Movie 1). Note also the important decrease in the number of Tau-GFP cells after day 3, due to cell death.

Movie 3 **Sonic Hedgehog treatment**. E13 Tau-GFP cortical cells treated with SHH and infected with retroviruses carrying the gene for RFP. Note the massive increase in numbers of RFP cells as compared to control (Movie 1). Notice also the slight decrease in the number of Tau-GFP cells after day 3.
